# Flavonoid extract Kushenol a exhibits anti‐proliferative activity in breast cancer cells via suppression of PI3K/AKT/mTOR pathway

**DOI:** 10.1002/cam4.4993

**Published:** 2022-07-04

**Authors:** Tao Liu, Jinhua Gong, Guobin Lai, Yichao Yang, Xiaoan Wu, Xiuping Wu

**Affiliations:** ^1^ Oncology Department Zhangzhou Zhengxing Hospital Zhangzhou China; ^2^ Xiamen Institute of Union Respiratory Health Xiamen China; ^3^ Department of Breast Surgery Zhangzhou Zhengxing Hospital Zhangzhou China

**Keywords:** apoptosis, breast cancer, Kushenol A, PI3K/AKT/mTOR pathway, proliferation

## Abstract

**Background:**

Kushenol A is natural flavonoid extract discovered in recent years, with potential anti‐tumor activity. Its role in breast cancer is poorly understood.

**Methods:**

To investigate biological function of Kushenol A in breast cancer (BC), Cell Counting Kit‐8 assay, colony formation assay, flow cytometry, western blotting, qPCR analysis, and xenograft mouse model were performed.

**Results:**

We found that Kushenol A treatment reduced proliferative capability and induced G0/G1 phase cell cycle arrest and apoptosis of BC cells in a concentration‐dependent manner. Besides, Kushenol A treatment contributed to the upregulation of apoptosis‐related and cell cycle‐associated genes. In nude mice, Kushenol A administration repressed BC xenograft tumor growth. Mechanistically, phosphorylation levels of AKT and mTOR were markedly attenuated in Kushenol A‐treated BC cells; however, there were no significant differences in total AKT and mTOR expressions. Moreover, PI3K inhibitor combined with Kushenol A exhibited synergistic inhibitory activity on cell proliferation.

**Conclusions:**

Taken together, our findings suggested that Kushenol A suppressed BC cell proliferation by modulating PI3K/AKT/mTOR signaling pathway. Kushenol A may be a promising therapeutic drug for treating BC.

## Graphical abstract

Kushenol A suppresses proliferation and induces apoptosis of breast cancer cells by inhibiting PI3K/AKT/mTOR signaling pathway. Kushenol A combined with PI3K inhibitor has a synergistic inhibitory effect on BC cell proliferation. Kushenol A is a potential therapeutic agent for breast cancer treatment.

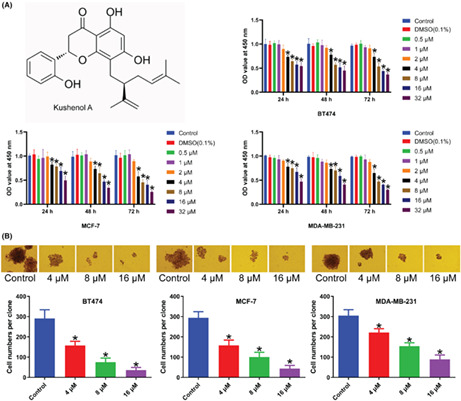

## 1 INTRODUCTION

Globally, the incidence of breast cancer (BC) ranks first among female patients with malignancy.^1^, ^2^ Currently, there have been great advances in the diagnosis and treatment of BC, leading to favorable outcomes in female patients with early‐stage BC.^3^, ^4^ However, survival is poor in patients with advanced BC, and current chemotherapeutic agents have limited efficacy and pronounced side effects.^5^, ^6^ Therefore, it is particularly important to develop new drugs for BC treatment.

Extracts and compounds extracted from herbal plants exhibit various bioactivities and play an increasingly important role in controlling BC with multiple targets and low toxic side effects.^7^, ^8^, ^9^ For example, curcumin is an acidic polyphenolic compound extracted from a variety of herbs with anti‐tumor activity.^9^, ^10^, ^11^ Previous works have found that curcumin has significant effects on apoptosis, autophagy, immunomodulation, and multidrug resistance.^12^, ^13^ Additionally, Citri Reticulatae Pericarpium extract hesperidin exhibited anti‐cancer activity in various cancer including BCs.^14^, ^15^, ^16^ Recently, Chen, Hao et al. have found that Kushenol Z and 14 other flavonoids including Kushenol A (compound 15) were isolated from Sophora flavescens, of which Kushenol Z and Kushenol A were potent to inhibit the proliferation of non‐small‐cell lung cancer (NSCLC) cells. Importantly, Kushenol Z facilitated NSCLC cell apoptosis via activation of mitochondrial apoptosis pathway.^17^

Kushenol A, a monomeric flavonoid,^18^, ^19^ was previously shown to be a non‐competitive tyrosinase inhibitor and has inhibitory effects on α‐glucosidase and β‐amylase.^18^, ^19^ Nevertheless, its biological function in BC remains to be explored. Considering the recent discovery that Kushenol Z displays cytotoxicity against NSCLC cells, and in respect to the chemical structure of Kushenol A and Kushenol Z, both have common parent nucleus, it provides us with an idea to explore biological function of Kushenol A in BC. The phosphatidylinositol 3‐kinase/protein kinase B/mammalian target of rapamycin (PI3K/AKT/mTOR) pathway plays a central role in cell growth, invasion, migration, apoptosis, and glucose metabolism.^20^ Inhibitors that target the PI3K/AKT/mTOR Pathway showed clinical benefit in patients with estrogen receptor (ER) positive BC and patients with triple‐negative breast cancer (TNBC).^21^, ^22^ Natural products were acting by the PI3K/Akt/mTOR signaling inhibition, showing their potency in cancer prevention and intervention.^23^ Regarding the specific molecular mechanisms of Kushenol A in BC cells, we believe that this question is interesting and merits further investigation.

Herein, we aim to understand Kushenol A effects on BC cells and identify the underlying mechanism by which Kushenol A exhibits antiproliferative activity via PI3K/AKT/mTOR pathway. A series of in vitro and in vivo experiments was performed. Our findings suggested that Kushenol A may be a promising therapeutic compound for BC treatment.

## 2 METHODS AND MATERIALS

### 2.1 Cell culture and reagent preparation

Human triple‐negative BC cells MDA‐MB‐231, the ER‐positive BC cells (MCF‐7 and BT474), were obtained from the Cell Bank of the Chinese Academy of Sciences (Shanghai, China). All Cells were cultured in DMEM medium containing 10% fetal bovine serum (FBS) (Gibco) and 1% penicillin/streptomycin (Gibco) in cell culture incubator at 37°C, with 5% CO_2_. Kushenol A (MedChemExpress, cat# HY‐N2278) was dissolved in DMSO (Sigma) and diluted in culture medium to final concentrations of 0.5, 1, 2, 4, 8, 16, and 32 μM, respectively. Cancer cells without Kushenol A treatment served as negative control. Tumor cells with 0.1% DMSO treatment were utilized as vehicle control.

### 2.2 Cell Counting Kit‐8 assay

BC cells (1 × 10^4^ cells/well) were seeded in 96‐well dishes and then subjected to Kushenol A for 24, 48 and 72 h, following incubation with Cell Counting Kit‐8 solution at 10% v/v for 2 h. Absorbance value (OD) at 490 nm was tested by microplate reader (Bio‐Rad Laboratories, Inc.). Cell proliferation and 50% inhibitory concentration (IC_50_) values were calculated. Adriamycin treatment was used as a positive control.

### 2.3 Colony formation assay

BC cells (300 cells/well) were seeded in 6‐well dishes and incubated with different concentrations of Kushenol A for 10 days. After the supernatant was discarded, the cells were fixed with methanol and stained with 0.1% crystal violet. Representative images were obtained under optical microscopy (Nikon). The number of cell clones in each well and cell numbers per clone were calculated.

### 2.4 Flow cytometry

For cell apoptosis assay, BC cells treated with Kushenol A for 48 h were harvested and incubated with Annexin V‐FITC and propidium iodide (PI) following manufacturer's instructions. For cell cycle assay, the adherent BC cells were collected and suspended in pre‐chilled 70% ethanol overnight at 4°C. After centrifugation, the cells were subjected to PI staining and RNAase A treatment for 20 min at 4°C, protected from light. A negative control and a blank control were set up. Samples were analyzed using CytoFLEX flow cytometer (Beckman Coulter). Data were quantified by Flow‐Jo software (Tree Star, Inc.).

### 2.5 qPCR analysis

Total RNA of BC cells was extracted using Trizol reagent (Invitrogen) following the manufacturers guidelines. cDNA was synthesized using HiScript III 1st Strand cDNA Synthesis Kit (+gDNA wiper) (Vazyme Biotech Co., Ltd, cat#R312‐01). qPCR was performed using Taq Pro Universal SYBR qPCR Master Mix (Vazyme Biotech Co., Ltd, cat#RQ712‐02) on ABI7500 Real‐Time PCR System (Applied Biosystems). Relative gene expression was normalized to GAPDH housekeeping gene and quantified by 2^−ΔΔ*Ct*^ method. Primer sequences are listed in Table 1.

**TABLE 1:** All primer sequences used in this study

Gene	Forward	Reverse
*GAPDH*	5′‐GCAAAGTGGAGATTGTTGCCAT‐3′	5′‐CCTTGACTGTGCCGTTGAATTT‐3′
*Bad*	5′‐CCCAGAGTTTGAGCCGAGTG‐3′	5′‐CCCATCCCTTCGTCGTCCT‐3′
*P21*	5′‐AATTGGAGTCAGGCGCAGAT‐3′	5′‐CGAAGAGACAACGGCACACT‐3′
*CyclinD1*	5′‐TGGAGCCCGTGAAAAAGAGC‐3′	5′‐TCTCCTTCATCTTAGAGGCCAC‐3′
*CDK4*	5′‐TTCGTGAGGTGGCTTTACTG‐3′	5′‐GATATGTCCTTAGGTCCTGGTCT‐3′
*Bax*	5′‐CCCGAGAGGTCTTTTTCCGAG‐3′	5′‐CCAGCCCATGATGGTTCTGAT‐3′
*Bcl‐2*	5′‐GGTGGGGTCATGTGTGTGG‐3′	5′‐CGGTTCAGGTACTCAGTCATCC‐3′
*Bcl‐xl*	5′‐GACTGAATCGGAGATGGAGACC‐3′	5′‐GCAGTTCAAACTCGTCGCCT‐3′
*caspase 3*	5′‐CATGGAAGCGAATCAATGGACT‐3′	5′‐CTGTACCAGACCGAGATGTCA‐3′
*caspase 9*	5′‐CTCAGACCAGAGATTCGCAAAC‐3′	5′‐GCATTTCCCCTCAAACTCTCAA‐3′
*PARP*	5′‐TCTGAGCTTCGGTGGGATGA‐3′	5′‐TTGGCATACTCTGCTGCAAAG‐3′
*CDK1*	5′‐AAACTACAGGTCAAGTGGTAGCC‐3′	5′‐TCCTGCATAAGCACATCCTGA‐3′
*CDK4*	5′‐TCAGCACAGTTCGTGAGGTG‐3′	5′‐GTCCATCAGCCGGACAACAT3′
*CDK6*	5′‐CCAGATGGCTCTAACCTCAGT‐3′	5′‐AACTTCCACGAAAAAGAGGCTT‐3′
*CDK7*	5′‐GGAGCCCCAATAGAGCTTATACA‐3′	5′‐TCCACACCTACACCATACATCC‐3′
*P53*	5′‐CAGCACATGACGGAGGTTGT‐3′	5′‐TCATCCAAATACTCCACACGC‐3′
*AKT*	5′‐TCCTCCTCAAGAATGATGGCA‐3′	5′‐GTGCGTTCGATGACAGTGGT‐3′
*mTOR*	5′‐TCCGAGAGATGAGTCAAGAGG‐3′	5′‐CACCTTCCACTCCTATGAGGC‐3′
*PI3K*	5′‐AAGAAGTTGAACGAGTGGTTGG‐3′	5′‐GCCCTGTTTACTGCTCTCCC‐3′

### 2.6 Western blotting analysis

BC cells were lysed with ice‐cold RIPA buffer supplemented with 1% PMSF. After centrifugation at 12,000 × g for 15 min at 4°C, supernatant containing proteins was collected. Protein concentration was assessed using BCA protein assay kit (Jiangsu KeyGEN BioTECH). Proteins were separated by SDS‐PAGE gel electrophoresis and transferred onto PVDF membranes. The membranes were blocked with 5% non‐fat milk for 2 h at room temperature (RT), incubated with primary antibodies overnight at 4°C, and treated with horseradish peroxidase‐labeled secondary antibody at RT for 2 h. Immunoblots were developed using BeyoECL Plus Kit (Beyotime Biotechnology). Data were quantified by Image J v1.8.0 software (NIH). Protein expression was normalized to GAPDH. All antibodies used in this study are listed in Table 2.

**TABLE 2:** Primary antibodies and corresponding secondary antibodies used in this study and their working dilutions

Antibodies	Manufacturer	Catalog no.	Working dilutions
GAPDH	Abcam	ab8227	1:5000
P21	Santa Cruz	sc‐6246	1:3000
CyclinD1	Abcam	ab226977	1:4500
CDK4	Abcam	ab137675	1:4500
PI3K	Abcam	ab32089	1:3500
Akt	Cell Signaling Technology	9272	1:3500
phosphorylated Akt (p‐Akt)	Abcam	ab131443	1:1500
cleaved‐caspase3	Cell Signaling Technology	9664	1:1500
cleaved‐PARP	Cell Signaling Technology	5625	1:1000
mTOR	Cell Signaling Technology	2972	1:1500
p‐mTOR	Cell Signaling Technology	5536	1:1500
caspase 3	Cell Signaling Technology	9662	1:3500
PARP	Cell Signaling Technology	9532	1:1500
caspase 9	Cell Signaling Technology	9508	1:1500
cleaved‐caspase 9	Cell Signaling Technology	20,750	1:1000
Bad	Cell Signaling Technology	9268	1:4500
Bax	Cell Signaling Technology	5023	1:4500
Bcl‐2	Cell Signaling Technology	15,071	1:4500
Bcl‐xl	Cell Signaling Technology	2764	1:4500
CDK1	Cell Signaling Technology	9116	1:1500
CDK6	Cell Signaling Technology	13,331	1:5000
CDK7	Cell Signaling Technology	2916	1:3500
P53	Cell Signaling Technology	9282	1:2500

### 2.7 Animal experiments

BALB/c female nude mice (weighing 18–20 g, 6 weeks old) were obtained from Cyagen Biosciences Inc. Nude mice were housed in an SPF‐grade laboratory animal center at 23–26°C and 35% relative humidity. All animal experiments were approved by the Animal Ethics Committee of the Zhangzhou Zhengxing Hospital. BC cells were adjusted with PBS to 2 × 10^7^ cells/ml with PBS and subcutaneous inoculation (2 × 10^6^ tumor cells per nude mouse) was performed. Nude mice and tumor growth were monitored daily (*N* = 5 mice per group). Body weight of the nude mice was tested weekly. Upon subcutaneous tumor nodules reaching a diameter of approximately 4 mm, Kushenol A was administered by gavage once a day for 2 weeks according to the experimental protocol. Tumor size was monitored every 3 days by vernier calipers and calculated as follows: 0.5 × length × width.^2^ After drug discontinuation, nude mice were sacrificed under anesthesia with isoflurane. Tumor nodules were peeled off and weighted to calculate tumor volume.

### 2.8 Statistics

Statistics were performed using SPSS 21.0 software (SPSS Inc.). One‐way analysis of variance (ANOVA) with Tukey's test was used to determine significant differences among multiple groups. Data were represented as mean ± standard deviation (SD). *p* < 0.05 was considered significant.

## 3 RESULTS

### 3.1 Kushenol A suppresses proliferation of BC cells

**FIGURE 1:**
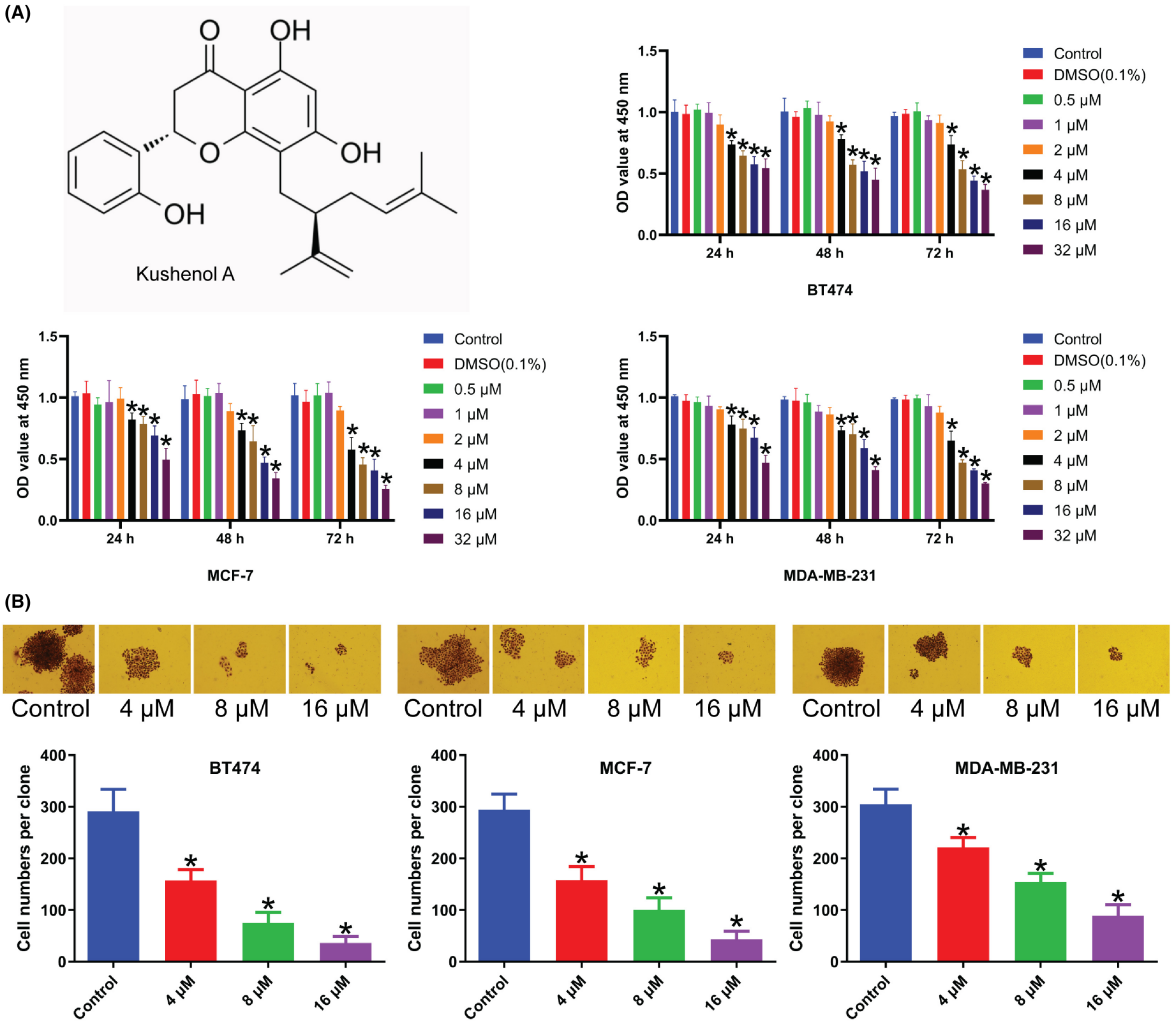
Different concentrations of Kushenol A affect BC cell proliferation. (A) Structural formula of Kushenol A (upper panel); cell viability of BT474, MCF‐7, and MDA‐MB‐231 cells. (B) Clone formation of BT474, MCF‐7, and MDA‐MB‐231 cells. *p < 0.05 by one‐way ANOVA and Tukey's test. BC, breast cancer.

To explore biological activity of Kushenol A on cell proliferative capacity, functional experiments were performed. We found that 0.5–2 μM of Kushenol A had no significant effects on proliferative ability of BC cells. 4–32 μM of Kushenol A suppressed BC cell proliferation in time‐ and concentration‐dependent manners compared to untreated control (Figure 1A). Besides, 0.1% DMSO as negative control had no effect on cell behavior. Given that 4–32 μM Kushenol A treatment was detected to have significant effects on BC cell proliferation, this range of concentrations of Kushenol A was applied to subsequent experiments. Colony formation assay showed a decrease in cell number per clone after 10 days of Kushenol A treatment compared to the control (Figure 1B).

### 3.2 Kushenol A induces apoptosis of BC cells

**FIGURE 2:**
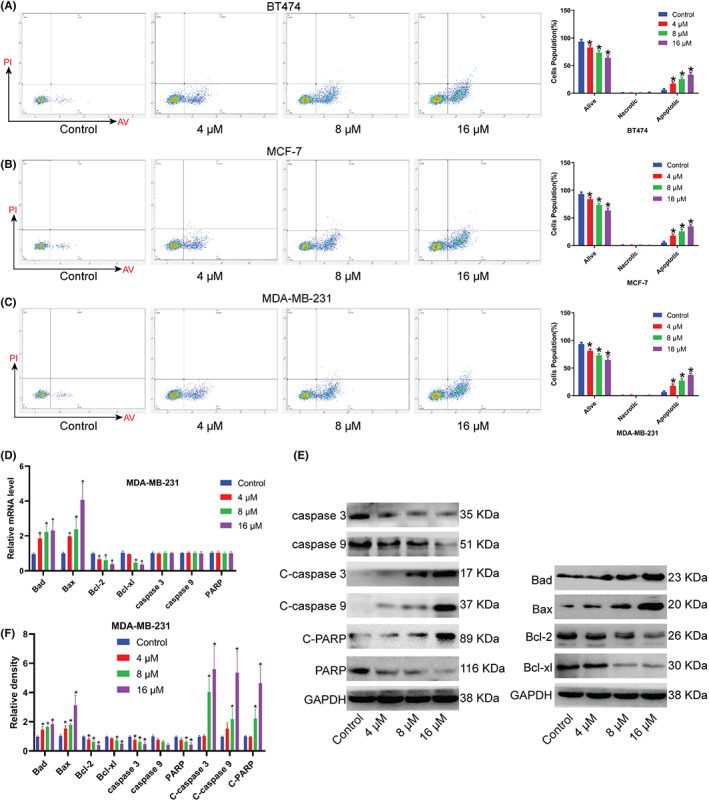
Different concentrations of Kushenol A promote BC cell apoptosis. (A–C) Effects of different concentrations of Kushenol A on apoptosis of (A) BT474, (B) MCF‐7, and (C) MDA‐MB‐231 cells. (D) The mRNA levels of Bad, Bax, Bcl‐2, Bcl‐xl, caspase 3, caspase 9, and PARP in MDA‐MB‐231 cells treated with different concentrations of Kushenol A for 48 h. (E) Representative immunoblots. (F) Quantification of protein expressions. *p < 0.05 by one‐way ANOVA and Tukey's test. BC, breast cancer.

Next, we explored the effect of Kushenol A on BC cell apoptosis. It was observed that Kushenol A caused cell apoptosis in a dose‐dependent manner compared with untreated control (Figure 2A–C). Meanwhile, mRNA levels of Bax and Bad were significantly upregulated, whereas Bcl‐2 and Bcl‐xl expression were down‐regulated in Kushenol A‐treated MDA‐MB‐231 cells compared to untreated control (Figure 2D). There were no significant differences in mRNA levels of caspase 9, caspase 3, and PARP in Kushenol A‐treated BC cells. Besides, western blotting analysis indicated that Kushenol A resulted in increased expression of pro‐apoptotic proteins (cleaved‐caspase 9, cleaved‐caspase 3, cleaved‐PARP, Bax, and Bad), and decreased expression of anti‐apoptotic proteins (Bcl‐2 and Bcl‐xl) in a dose‐dependent manner. Kushenol A treatment had no significant effects on protein levels of caspase 9, caspase 3, and PARP (Figure 2E,F).

### 3.3 Kushenol A causes G0/G1 phase cycle arrest in BC cells

**FIGURE 3:**
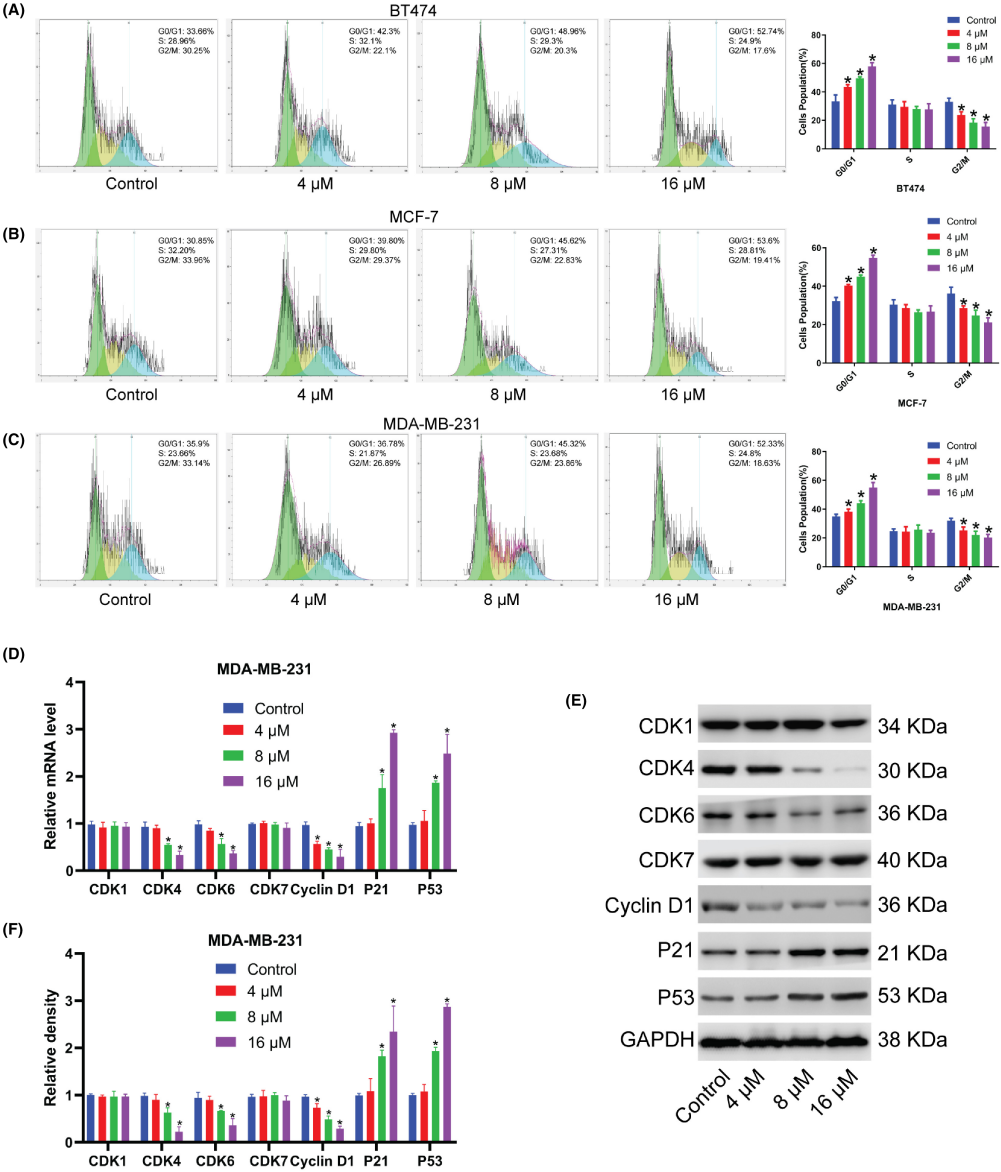
Different concentrations of Kushenol A treatment block cell cycle progression of BC cells. (A–C) Effects of different concentrations of Kushenol A on cell cycle progression of (A) BT474, (B) MCF‐7, and (C) MDA‐MB‐231 cells. BC cells were subjected to Kushenol A for 48 h. (D) The mRNA levels of CDK1, CDK4, CDK6, CDK7, Cyclin D1, P21, and P53 in MDA‐MB‐231 cells treated with Kushenol A for 48 h. (E) Representative immunoblots. (F) Statistical analysis of protein expressions. *p < 0.05 by one‐way ANOVA and Tukey's test. BC, breast cancer.

The effects of different concentrations of Kushenol A (4, 8, and 16 μM) on cell cycle progression were evaluated using PI staining at 48 h after treatment. Compared with untreated control, Kushenol A treatment contributed to G0/G1 phase cell cycle arrest in a dose‐dependent manner (Figure 3A–C). No significant effect on cancer cells at the S phase was observed. Mechanistically, Kushenol A treatment significantly reduced the expression of cyclin‐dependent kinases CDK4, CDK6, and Cyclin D1, and promoted the expression of cyclin‐dependent kinase (cdk) inhibitor P53 and P21 at both translational and transcriptional levels (Figure 3D–F). CDK1 and CDK7 expression levels in BC cells were not affected by Kushenol A.

### 3.4 Kushenol A inhibits BC xenograft tumor growth

**FIGURE 4:**
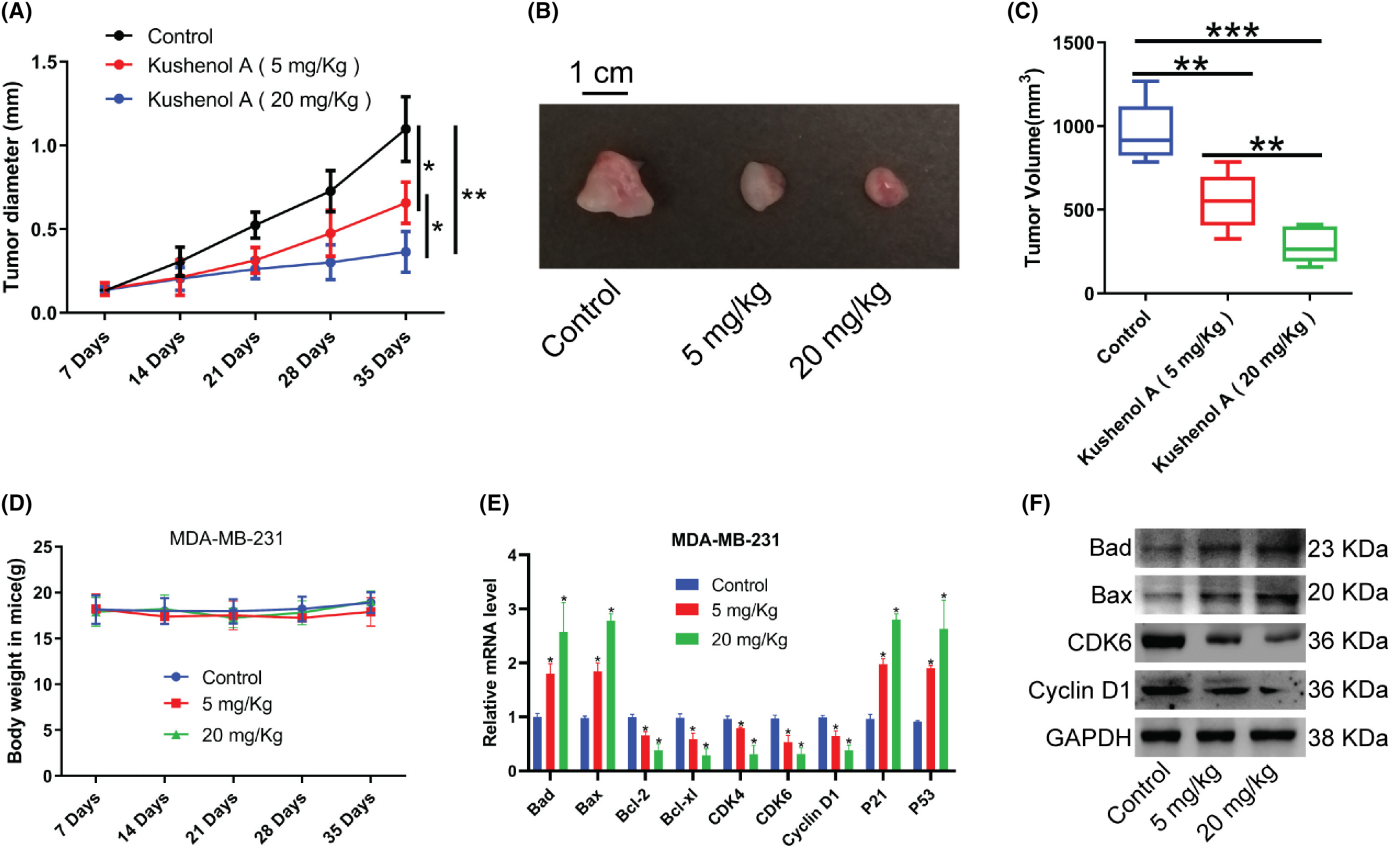
Kushenol A administration represses xenograft tumors growth. (A) Effects of two doses of Kushenol A administration on tumor diameters at different time points. (B) Representative images of breast cancer xenograft tumors. (C) Quantification of tumor volumes at 35 days. (D) Body weight of mice. (E) The mRNA levels of Bad, Bax, Bcl‐2, Bcl‐xl, CDK4, CDK6, Cyclin D1, P21, and P53 in xenograft tumors. (F) The protein expressions of Bad, Bax, Bcl‐2, CDK6, and Cyclin D1 in xenograft tumors. *p < 0.05, **p < 0.01, and ***p < 0.001 by one‐way ANOVA and Tukey's test. BC, breast cancer.

To further determine anti‐tumor activity of Kushenol A on BC cells, we established subcutaneous xenograft mouse model of BC. Data confirmed that Kushenol A significantly restrained the BC cell proliferation. The inhibitory effect was enhanced by increasing the Kushenol A dose (Figure 4A–C). Body weight of nude mice was not changed during experiments (Figure 4D). In addition, consistent with our observations in vitro, mRNA and protein levels of the aforementioned genes were significantly altered in xenograft tumor tissues (Figure 4E,F).

### 3.5 Kushenol A represses PI3K/AKT/mTOR pathway

**FIGURE 5:**
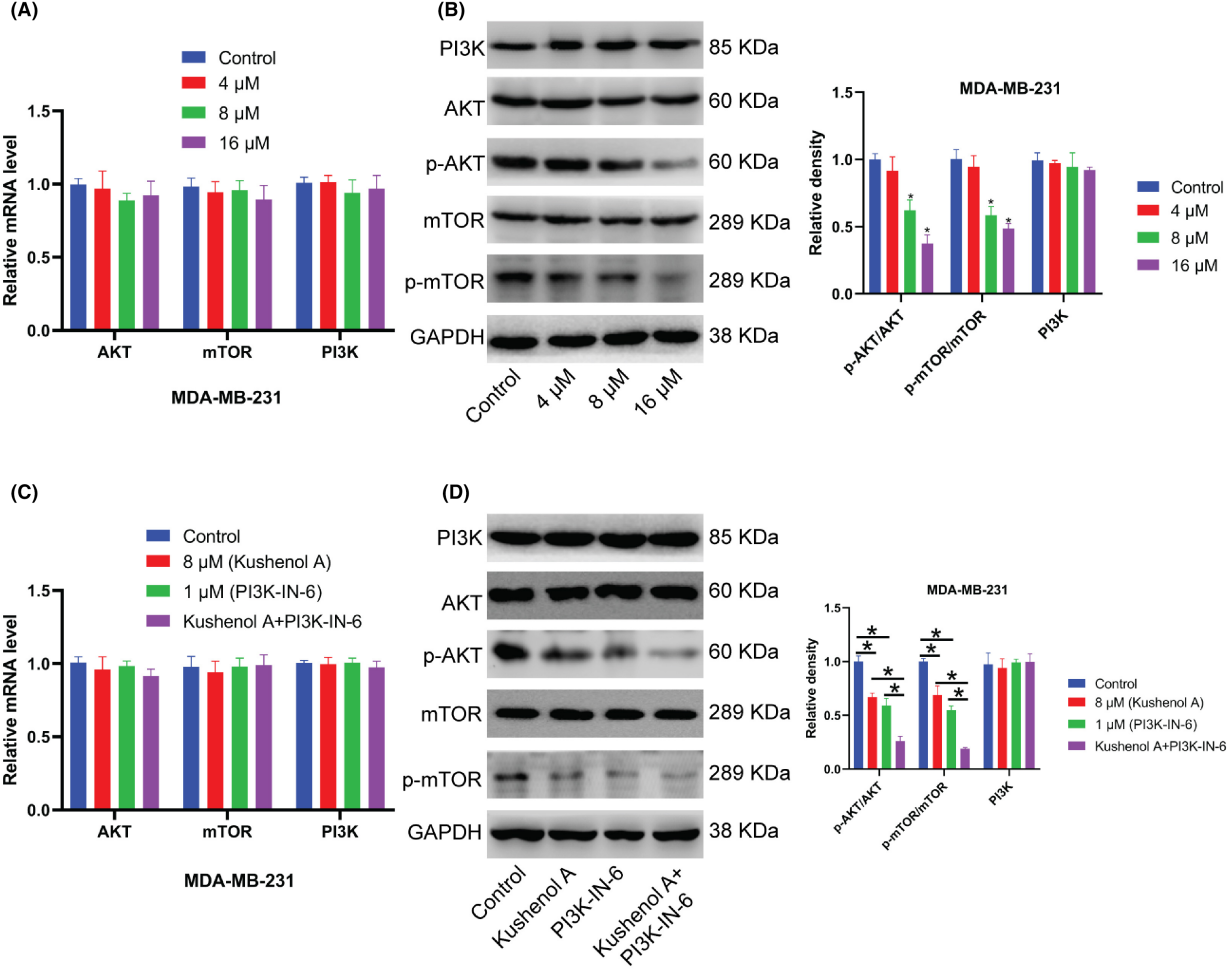
Kushenol A treatment inactivates PI3K/AKT/mTOR pathway in BC cells. (A) The mRNA levels of AKT, mTOR and PI3K in MDA‐MB‐231 cells that were treated with Kushenol A for 48 h. (B) Representative immunoblots and quantification of protein expressions. (C) Effects of 8 μM Kushenol A treatment alone or in combination with 1 μM PI3K inhibitor PI3K‐IN‐6 for 48 h on mRNA levels of AKT, mTOR and PI3K in MDA‐MB‐231 cells. (D) Representative immunoblots and quantification of protein expressions. *p < 0.05 by one‐way ANOVA and Tukey's test. BC, breast cancer.

**FIGURE 6:**
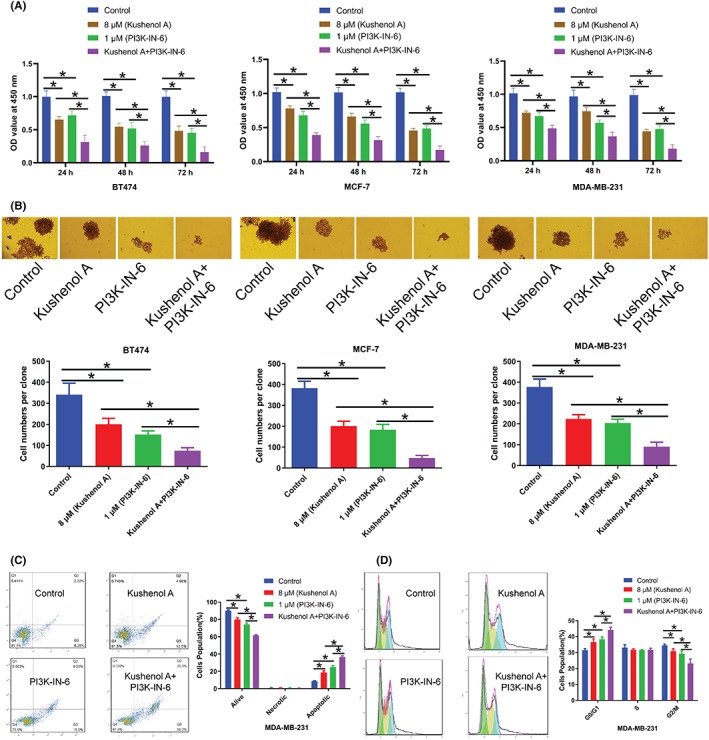
Kushenol A treatment alone or combined with PI3K inhibitor PI3K‐IN‐6 decreases proliferative ability of BC cells. (A) Cell viability of BT474, MCF‐7, and MDA‐MB‐231 cells that were subjected to 8 μM Kushenol A or/and 1 μM PI3K‐IN‐6 for 48 h was evaluated by Cell Counting Kit‐8 assays. (B) Colony formation capabilities of BC cells that were treated with 8 μM Kushenol A or/and 1 μM PI3K‐IN‐6 for 48 h was assessed by colony formation assays. (C) Cell apoptosis and (D) cell cycle distribution of MDA‐MB‐231 cells that were treated with 8 μM Kushenol A or/and 1 μM PI3K‐IN‐6 for 48 h. *p < 0.05 by one‐way ANOVA and Tukey's test. BC, breast cancer.

To further explore the underlying mechanism by which Kushenol A inhibited BC cell proliferation, the activation of PI3K/AKT/mTOR pathway was examined after Kushenol A treatment. Data uncovered that Kushenol A treatment reduced phosphorylation of AKT and mTOR in a dose‐dependent manner. Total AKT and total mTOR were not changed (Figure 5A,B). Moreover, PI3K inhibitor PI3K‐IN‐6 treatment combined with Kushenol A further attenuated phosphorylation of AKT and mTOR in BC cells, with no notable effect on PI3K expression (Figure 5C,D). The cotreatment displayed synergistic inhibitory effects on proliferative ability of BC cells, while Kushenol A synergized with PI3K‐IN‐6 to promote cell apoptosis. (Figure 6). Overall, these findings suggested that Kushenol A produced antitumor effects by affecting PI3K/AKT/mTOR signaling axis.

## 4 DISCUSSION

Breast cancer is the most common cancer among women.^24^ Although therapeutic strategies have been greatly developed, the clinical effectiveness of current chemotherapeutic drugs needs to be improved.^6^, ^24^ To improve the outcome of patients with BC, it is particularly important for the development of new therapeutic agents. In this study, Kushenol A attenuated proliferative capacity and promoted apoptosis of BC cells via the PI3K/AKT/mTOR pathway inhibition. Furthermore, Kushenol A and PI3K inhibitor PI3K‐IN‐6 coordinately repressed BC cell growth. Kushenol A exhibited anti‐tumor activity and holds promise as a pharmacologic treatment for BC.

Dysfunction of cell proliferation is critically important in tumorigenesis and is one of the distinguishing features of tumors.^25^, ^26^ Cell cycle progression is an essential process for proliferation,^27^, ^28^ of which CDK4/CDK6/cyclin‐D1 complex regulates the G1 and G1/S phase transitions. Pharmacologic inhibitors of CDK4/CDK6 result in G1 phase cell cycle arrest, suppressing cancer cell proliferation.^29^, ^30^ Several specific CDK4/CDK6 inhibitors have been developed and have achieved good preclinical and clinical therapeutic results.^31^, ^32^, ^33^ In this study, we found that Kushenol A treatment markedly downregulated CDK4 and CDK6 expressions as well as other cell cycle‐related genes. And Kushenol A caused G0/G1 phase cell cycle arrest, suggesting that the effect of Kushenol A in inhibiting BC cell proliferation is at least partially mediated by repressing cell cycle progression.

Cell apoptosis is an important regulator during tumor development.^34^, ^35^ Apoptosis is induced by multiple signaling pathways, culminating in cell death through the activation of apoptosis‐related proteins.^36^ Caspase 9, the initiator of apoptotic process, triggers a series of sequential cascade reactions, leading to the activation of caspase 3 which executes cell apoptosis.^37^, ^38^ Activated caspase 3 has been considered a reliable marker for cell apoptosis.^39^ Here, cleaved‐Caspase 9 and cleaved‐caspase 3 were significantly up‐regulated in BC cells subjected to Kushenol A treatment. This is one of the reasons why Kushenol A caused cancer cell apoptosis.

Considering that both cell cycle and apoptosis are cellular phenotypes following Kushenol A treatment, we then explored molecular mechanisms underlying these effects and found that Kushenol A regulated PI3K/AKT/mTOR pathway. PI3K/AKT/mTOR pathway is aberrantly activated during tumorigenesis and significantly affects malignant phenotypes of cancer cells, as well as chemotherapy resistance.^40^, ^41^ Inhibiting PI3K/AKT/mTOR pathway is able to slow down tumor progression.^42^, ^43^ By combining Kushenol A and PI3K inhibitor, it was confirmed that proliferative capacity of BC cells was further reduced, indicating that Kushenol A inhibited BC cell proliferation and facilitated apoptosis via the PI3K/AKT/mTOR pathway. Consistent with our results, Kushenol Z, the chemical analogue of Kushenol A, regulates mTOR pathway by suppressing phosphodiesterase and Akt activity to cause apoptosis in NSCLC cells.^17^ Similarly, flavonoids apigenin and vitexin induced apoptosis in HepG2 and A549 cells, respectively, both of which exert effect through PI3K/AKT/mTOR pathway.^44^, ^45^ These bioactive compounds derived from natural products target the PI3K‐Akt–mTOR signaling pathway for cancer prevention and intervention.^23^ In addition, it is worth noting that there are three BC cell lines that differ in genetic variants used in this study. Therefore, different responses from Kushenol A by the disparate signaling cascades will have existed. Regarding of the specific molecular mechanisms of Kushenol A in different types of BC cells, we believe that this question is interesting and merits further investigation.

In summary, we demonstrated that Kushenol A suppressed proliferation and induced apoptosis of BC cells via inhibition of the PI3K/AKT/mTOR signaling axis. Our findings provide evidence for Kushenol A as a promising therapeutic compound for BC treatment.

## CONFLICT OF INTEREST

The authors declare that they have no conflict of interest.

## ETHICS APPROVAL STATEMENT

Animal experiments were reviewed and approved by the Ethics Committee of Zhangzhou Zhengxing Hospital.

## Data Availability

All relevant data are within the article. The data that support the findings of this study are available from the corresponding author upon reasonable request.
